# Defining enteric bacterial pathogenesis using organoids: *Citrobacter rodentium* uses EspC, an atypical mucinolytic protease, to penetrate mouse colonic mucus

**DOI:** 10.1080/19490976.2025.2494717

**Published:** 2025-05-05

**Authors:** Yan Chen, Ashley Gilliland, Qiaochu Liang, Xiao Han, Hyungjun Yang, Jocelyn Chan, Dominique Lévesque, Kyung-Mee Moon, Parandis Daneshgar, François-Michel Boisvert, Leonard Foster, Wesley F. Zandberg, Kirk Bergstrom, Hong B. Yu, Bruce A. Vallance

**Affiliations:** a Division of Gastroenterology, Department of Pediatrics, University of British Columbia, Vancouver, BC, Canada; b Department of Immunology and Cell Biology, Université de Sherbrooke, Sherbrooke, Canada; c Department of Biochemistry and Molecular Biology, Michael Smith Laboratories, Life Sciences Institute, University of British Columbia, Vancouver, BC, Canada; d Department of Chemistry, University of British Columbia, Kelowna, BC, Canada; e Department of Biology, University of British Columbia, Kelowna, BC, Canada; f Department of Microbiology, Molecular Genetics and Immunology, University of Kansas Medical Center, Kansas, USA

**Keywords:** Enteric pathogens, EspC, air-liquid interface, colonoids, mucus

## Abstract

Enteric bacterial pathogens pose significant threats to human health; however, the mechanisms by which they infect the mammalian gut in the face of daunting host defenses remain to be fully defined. For the attaching and effacing (A/E) bacterial family member and murine pathogen *Citrobacter rodentium*, its virulence strategy appears to involve penetration of the colonic mucus barrier to reach the underlying epithelium. To better define these interactions, we grew colonoids under air-liquid interface (ALI) conditions, producing a thick mucus layer that mimicked *in vivo* mucus composition and glycosylation. *C. rodentium*’s penetration of ALI-derived mucus was dramatically enhanced upon exposure to sialic acid, in concert with the secretion of two serine protease autotransporter of *Enterobacteriaceae* (SPATE) proteins, Pic and EspC. Despite Pic being a class II SPATE, and already recognized as a mucinase, it was EspC, a class I SPATE family member, that degraded ALI-derived mucus, despite class I SPATEs not previously shown to possess mucinase activity. Confirming this finding, *E. coli* DH5α carrying a plasmid that expresses *C. rodentium*-derived EspC was able to degrade the mucus. Moreover, recombinant EspC alone also displayed mucinolytic activity in a dose-dependent manner. Collectively, our results reveal the utility of ALI-derived mucus in modeling microbe-host interactions at the intestinal mucosal surface, as well as identify EspC as an atypical class I SPATE that shows significant mucinolytic activity toward ALI-derived mucus.

## Introduction

Most enteric bacterial pathogens target their host’s intestinal epithelium, with an array of published studies defining the mechanisms they use to subvert intestinal epithelial cell (IEC) functions and successfully parasitize their hosts.^1,2^ In contrast, the virulence strategies they utilize to overcome luminal defenses, and ultimately reach the epithelium are poorly defined. In part, this reflects the human specificity of most clinically important bacterial pathogens, and their inability to infect the intestines of laboratory animal species.^3^ Over the last two decades, many advances made in the field of enteric bacterial pathogenesis have come from the study of *Citrobacter rodentium*, a bacterial pathogen that readily infects the colons of mice, using virulence factors similar to those expressed by the clinically important attaching and effacing (A/E) pathogens enteropathogenic *Escherichia coli* (EPEC) and enterohemorrhagic *E. coli* (EHEC).^1,4^

A/E pathogens are known for their intimate attachment to IECs via their type III secretion system (T3SS),^5,6^ but their specific actions prior to this phase of infection are unclear. Previous studies have shown that *C. rodentium* initially colonizes the gut lumen, where commensal microbiota can outcompete it for both space and nutrients.^7,8^ *C. rodentium*’s success as a pathogen thus depends on its ability to escape the lumen and reach its preferred IEC-adherent niche. This requires *C. rodentium* to circumvent the colonic mucus layer, a physiochemical barrier that effectively segregates commensal microbes away from the colonic epithelium. Colonic mucus is predominantly composed of the heavily glycosylated mucin Muc2, which is apically secreted into the lumen by goblet cells, where it hydrates to form a complex matrix containing many other IEC-derived constituents.^9,10^ The importance of Muc2 in host defense was previously demonstrated by the exaggerated susceptibility of *Muc2* deficient (^−/−^) mice to *C. rodentium* infection.^11^ Interestingly, the membrane mucin Muc17, which forms the glycocalyx layer, is also shown to protect the host against *C. rodentium* infection.^12^ Despite the critical role played by mucus as an intestinal mucosal barrier, many commensal bacteria forage on the outer regions of the mucus layer to acquire nutrients. They typically use glycoside hydrolase (GH) enzymes to cleave terminal monosaccharides from Muc2, while leaving the underlying mucus barrier intact.^9,13^ Many enteric pathogens are able to catabolize these monosaccharides, facilitating their rapid expansion within the gut and their ability to attach or invade IECs.^14,15^

Serine protease autotransporters of *Enterobacteriaceae* (SPATEs) are a superfamily of virulence proteins secreted by many enteric bacterial pathogens.^16,17^ SPATEs are divided into two groups, class I and class II, based on differences in their structure and functional properties. Class I SPATEs, including EspC, Pet, Sat and SigA, exhibit enterotoxic and cytotoxic activity toward IECs,^18–21^ while class II SPATEs, such as Pic, Hbp and EatA, possess O-glycoprotease activity and are often involved in mucosal colonization.^22–24^ Some pathogens express both class I and II SPATEs. For instance, *C. rodentium* carries at least three SPATEs, namely EspC, Pic, and AdcA (class II), although only EspC and Pic can be detected in *in vitro* cultures.^25^ We previously demonstrated that exposure to sialic acid, a monosaccharide derived from the Muc2 mucin, enhances the ability of *C. rodentium* to degrade bovine submaxillary mucin (BSM) via Pic and adhere to IECs via EspC.^26^ Despite these findings, our inability to properly model colonic mucus left it unclear whether *C. rodentium* uses Pic and/or EspC to breach the mucus barrier.

Historically, it has been very challenging to create a relevant mucus barrier *in vitro*, as few IEC lines produce Muc2.^27,28^ However, 2-dimensional (2D) monolayer models derived from 3D intestinal organoids have been recently used to study microbe-mucus interactions.^29^ Mucus layer formation can be induced by growing confluent organoid monolayers in the presence of differentiation media.^30^ Using this method, Enteroaggregative *E. coli* Pic was shown to degrade mucins secreted by a human colonoid-derived monolayer.^31^ Another way to generate mucus is through air-liquid interface (ALI) culturing, where the apical side of the monolayer is exposed to air while the basal side remains submerged in growth media.^32,33^ This approach promotes oxidative phosphorylation within IECs, leading to increased cellular differentiation, goblet cell formation, and Muc2 secretion, as well as the formation of an overlying mucus layer.^34^ While ALI-derived mucus has been suggested to closely mimic *in vivo* mucus,^35^ its glycosylation status, as well as protein composition has not been determined. Despite this, it has been successfully used to study several pathogens, including parasites and viruses.^36–38^

In this study, we generated mouse colonoid ALI cultures and characterized the resulting mucus using proteomic and glycomic approaches, demonstrating its significant similarity to *in vivo* mucus. We also found that sialic acid dramatically enhanced the ability of *C. rodentium* to degrade and penetrate ALI-derived mucus. In contrast to prior analysis with BSM, EspC proved more important than Pic in mediating the degradation of the ALI-derived mucus. The mucinolytic activity of *C. rodentium* EspC was confirmed through expressing EspC heterologously in *E. coli* DH5α as well as the testing of recombinant EspC. Taken together, our findings demonstrate the utility of ALI-derived mucus in the study of pathogen-mucus interactions at the intestinal mucosal surface. Moreover, we determine that EspC functions as an atypical class I SPATE that exhibits significant mucinolytic activity, playing an important role in *C. rodentium*’s pathogenic strategy to reach the host’s colonic epithelium.

## Materials and methods

### Ethics statement

All mouse experiments were conducted in compliance with protocols approved by the University of British Columbia’s Animal Care Committee (A23–0204) and were strictly adherent to the guidelines of the Canadian Council on Animal Care.

### Mouse strain

Six to ten-week-old *Muc2*^+/+^ and *Muc*2^−/−^ mice were obtained from Dr. Anna Velcich (Albert Einstein Cancer Centre) and bred at the BC Children’s Hospital Research Institute (BCCHRI) animal care facility. Mice were maintained in sterilized, filter-topped cages, and provided with autoclaved food and water under specific pathogen-free (SPF) conditions. At six to ten weeks of age, both male and female mice were euthanized and their distal colonic tissues were used for organoid growth.

### Generation of mouse colonoids and maintenance

Mouse colonoids were grown as previously described.^39^ Briefly, colonic crypts were isolated from distal colons, washed, and pelleted before being suspended in Matrigel (Corning 356231) at the final concentration of 5 mg/mL. A volume of 30 µL of the mixture was plated in 24 well plates. Once the Matrigel solidified, 500 µL of organoid media was added to each well. The organoid growth medium consisted of 50% Advanced DMEM/F12 (Gibco 12634010) supplemented with PenStrep (Gibco 15140122), GlutaMAX (Gibco 35050061) and HEPES (Gibco 15630080), combined with 50% WRN conditioned medium supplemented with N2 (Invitrogen 17502048), B27 (Invitrogen 17504044), N-acetylcystine (Sigma-Aldrich, A9165), nicotinamide (Sigma-Aldrich, N0636), mEGF (Invitrogen, PMG8043), A83–01 (Tocris, 2939), SB 202190 (Sigma-Aldrich, S7067) and Rock inhibitor Y-27632 (AbMole, M1817). Plates were incubated at 37°C with 5% CO_2_. The medium was changed every two to three days and the colonoids were passaged every five to seven days.

### ALI growth and maintenance

Mouse ALI cultures were established as previously described.^32^ In brief, mouse colonic spheroids were dissociated into single cells by TrypLE Express reagents (Gibco 12605036). A total of 300,000 cells suspended in 200 µL monolayer medium were seeded onto Transwells (Corning, C3470) pre-coated with 5% Matrigel (Corning) and incubated at 37°C for two hours. Subsequently, 500 µL of monolayer medium was added to the bottom chambers of the Transwells. Monolayer medium consisted of 50% Advanced DMEM/F12 and 50% WRN, supplemented with PenStrep, GlutaMAX and HEPES, N2, B27, mEGF, and Y-27632. After 7 days of growth, the medium in the upper chamber was removed and the cells were grown in ALI conditions with the medium in the bottom chamber for up to an additional 21 days. The medium was changed every two to three days.

### Immunofluorescence and lectin staining

ALI cultures were fixed using 200 µL of 4% PFA in the upper and 500 µL in the bottom chambers for 30 minutes at room temperature. The fixed ALI cultures were embedded in 3% agarose gel and sent to UBC’s Animal Care Services (ACS) Diagnostic & Research Histology Laboratory for paraffin processing, embedding, and sectioning. Paraffin-embedded tissue sections (5 μm thick) were deparaffinized by heating in hot steam for 20 min, followed by four rounds of soaking in xylene. The sections were then rehydrated using sequential treatment with 100% (2 rounds), 95%, and 70% ethanol, followed by final rinsed in distilled water, with each step lasting 3 minutes. Antigen retrieval was performed by boiling the deparaffinized sections in sodium citrate buffer (pH 6.0) for 35 minutes. The ALI sections were then permeabilized with the permeabilization buffer (0.1% Triton-X100 and 0.05% Tween 20 in PBS) for 15 minutes, followed by blocking for 1 hour in blocking buffer (2% donkey serum, 0.1% Triton-X100, and 0.05% Tween 20 in PBS). The sections were stained with the following primary antibodies and lectins: rabbit anti-LPS (Bio-Rad, OBT0986) for *C. rodentium*, rabbit anti-Muc2 (Boster, A01212; Novus Biologicals, NBP1–31231), UEA-1 (Vector Laboratories, FL-1061), MALII (Vector Laboratories, B-1265-1) for mucus, and mouse anti-E-cadherin (BD Transduction Laboratories 610182) for IECs. The secondary antibodies for staining include: Alexa Fluor 568-conjugated donkey anti-rabbit IgG and Alexa Fluor 647-conjugated donkey anti-mouse IgG (Life Technologies). Slides were then mounted with ProLong Gold Antifade reagent containing DAPI (Invitrogen, P36931). Imaging was performed using a Zeiss AxioImager microscope, and images were captured with an AxioCam HRm camera operating through Zen software and analyzed by ImageJ.^40^

### Proteomics analysis of the ALI-derived mucus

On day 21, ALI-derived mucus was collected by adding 100 µL PBS to the Transwell and allowing it to soak for 10 minutes. The collected mucus was sent to the Proteomics Facility at the Université de Sherbrooke for sample preparation and mass spectrometry (MS) analysis, as described previously.^41^ Briefly, three volumes of denaturing buffer (8 M urea, 1 M NH_4_HCO_3_, 20 mm HEPES, pH 7.5) were added to the mucus and the mixture was sonicated to reduce viscosity. A total of 75 µg of protein extract was reduced with DTT, alkylated with chloroacetamide (Sigma-Aldrich), and digested with trypsin (Thermo Fisher Scientific). Peptides were purified using C18 columns, concentrated, and resuspended in formic acid buffer. For data-independent acquisition (DIA) liquid chromatography-mass spectrometry (LC – MS) analysis, 250 ng of peptides from each sample were loaded onto a trap column and separated on an analytical C18 column using a gradient of acetonitrile. The peptides were analyzed on a TimsTOF Pro mass spectrometer and data were acquired using diaPASEF mode. The raw data was analyzed with DIA-NN software (version 1.8.1), as described previously,^42^ with the Uniprot mouse proteome database.

### Glycomics analysis of the ALI-derived mucus

ALI-derived mucus was collected on day 21 and freeze-dried for glycan extraction and LC-MS, as previously described.^43^ In brief, mucin O-glycans were released using the Carlson reductive β-elimination method and then neutralized. The neutralized reaction was applied to preconditioned ENVI-Carb solid-phase extraction cartridges. Glycan separation was performed using a Hypercarb high-performance liquid chromatography (HPLC) column. LC-MS data acquisition and analysis were carried out using MassHunter Workstation software (Agilent Technologies). For each sample, the peak areas of all detected glycans were normalized to the total glycan area. Glycan data were analyzed and visualized in RStudio (R version 4.3.1). The relative abundances of various glycan groups (e.g., neutral, fucosylated, and sialylated) were quantified.

### Bacterial strains and culture conditions

*C. rodentium* strain DBS100 (streptomycin-resistant) was used as the WT bacterial strain. The Δ*pic*, Δ*espC* and Δ*espC*Δ*pic* double mutant (ΔΔ) were from a previous study.^43^ Bacteria were cultured either on Luria-Bertani (LB) agar plates supplemented with 100 μg/mL streptomycin or in LB broth with shaking at 200 rpm at 37°C overnight.

### C. rodentium *infection of ALI colonoid monolayers*

*C. rodentium* overnight cultures grown in LB were subcultured at 1:20 ratio into 5 mL Dulbecco’s modified Eagle’s medium (DMEM) supplemented with either 0.45% (w/v) glucose or sialic acid. The cultures were incubated in a tissue culture incubator at 37°C with 5% CO_2_ for 3–5 hours to reach mid-exponential-phase growth. *C. rodentium* of an MOI 25 in a volume of 30 µL was layered onto the mucus surface of ALI culture for up to 10 hours.

### RNA extraction and qPCR

A total of 400 µL of Trizol (Invitrogen) was added to the top chamber of ALI cultures, two times. Monolayer cells on the Transwells were scraped and lysed at room temperature for 15 minutes. RNA was extracted using Direct-zol RNA Micro-Prep Kit (Zymo Research) according to the manufacturer’s instructions. Total RNA was quantified using a NanoDrop 1000 Spectrophotometer (Thermo Fisher Scientific). For cDNA synthesis, 500 ng of RNA was reverse-transcribed using 5× All-In-One RT (abm), and the resulting cDNA was diluted 1:5 in RNase/DNase-free water. Quantitative PCR (qPCR) was performed using a Bio-Rad CFX connect Real-time PCR detection system. Data analysis and quantitation were carried out with CFX Maestro software (Bio-Rad). *Muc2* gene expression was normalized to the housekeeping gene *Rplp0* (Ribosomal protein lateral stalk subunit) using the 2^−ΔCt^ method for relative transcript levels. mRNA expression levels were normalized to the control group using the 2^−ΔΔCt^ method.

### *Protein secretion of* C. rodentium, *mucus degradation assay, and western blot*

Protein secretion was induced in 5 mL DMEM supplemented with either 0.45% (w/v) glucose or sialic acid as described previously.^43^ Equal volumes of culture (normalized by OD_600_) were centrifuged to pellet the cells, and the supernatant was filtered through a 0.22-µm filter to remove any remaining bacterial cells. The filtered supernatants were then concentrated to approximately 50 µL using Amicon Ultra 4 filters with 50-kDa cutoff (Millipore). For the mucus degradation assay, 21 µL of the concentrated protein sample were incubated overnight at 37°C with 0.2 µg of ALI-derived mucus. The degradation reaction was reduced with 5% β-mercaptoethanol, resolved on a 3–8% Tris-acetate gradient gel (NuPAGE, Invitrogen) and transferred to PVDF membrane using an iBlot system (ThermoFisher). Muc2 degradation products were detected by immunoblotting using an anti-Muc2 rabbit polyclonal antibody (Novus Biologicals, NBP1–31231) (1:1,000) which targets an epitope on the C terminus of mucin 2 from both human and mouse origin.

### *Gene synthesis and expression in* E.Coli *DH5α*

*CrespC*, *Crpic*, EPEC*espC* genes were synthesized and cloned into pBAD30 (Amp^r^) vector using SacI and XbaI, and transformed them into *E.coli* DH5α (GenScript Biotech). We also performed site-directed mutagenesis on *CrEspC*, changing the conserved serine (AGC) in the protease domain GD*S*GS into isoleucine (ATC). *E.coli* DH5α strains carrying either empty vector (pBAD30) or plasmids with expressed genes (pBAD30-*CrespC*, pBAD30-*Crpic*, pBAD30-*CrespC-S251I*, pBAD30-EPEC*espC*), were grown in LB supplemented with 100 µg/mL of Ampicillin overnight, subcultured in LB (1:50) till OD_600_ of 0.6–0.8. This was followed by the addition of 0.2% arabinose for 4 h, to induce the expression of proteins of interest. Culture is pelleted by centrifugation, and the supernatant was filtered through a 0.22-µm filter to remove bacterial cells. 21 µL of supernatant was used for mucus degradation assay followed by the visualization of degraded products by western blot.

### Coomassie staining and MS analysis of degraded Muc2 band

The gel was fixed in a fixation solution containing 40% ethanol, 10% acetic acid, 50% deionized H_2_O for 30 min, followed by washing with ddH_2_O twice. The gel was then incubated in the staining solution (0.12% Coomassie G-250, 10% ammonium sulfate, 10% o-phosphoric acid, and 20% methanol) for 2 hours. Excess stain and background were removed by successive washes with ddH_2_O. After the degraded mucus band was confirmed by western blot using anti-Muc2 antibody, the band at the molecular weight of approximately 225kDa was excised, reduced, alkylated, and subjected to enzymatic digestion using trypsin (New England Biolabs) for MS analysis, as described previously.^44^ Samples were analyzed on a TimsTOF Pro2 (Bruker Daltonics) MS coupled to liquid chromatography with Captive Spray ionization. PASEF mode was used for data-dependent acquisition. Data were searched using FragPipe v22.0 against the Uniprot mouse database (UP000000589) with strict trypsin specificity.^45^

### Recombinant protein expression, purification, and mucus degradation

The pelB signal sequence (MKYLLPTAAAGLLLLAAQPAMA), His-tag (6 histidine), CrEspC passenger domain and β-barrel domain were constructed and cloned into the plasmid pBAD30, followed by transformation into *E. coli* BL21 Star (DE3) strain for protein expression and purification (GenScript Biotech). Briefly, *E. coli* BL21 pBAD30 strain was inoculated in terrific broth supplemented with ampicillin (100 µg/mL). The medium was centrifugated and the supernatant was applied to a Ni column preequilibrated with equilibration buffer (50 mm Tris-HCl, 150 mm NaCl, 1 mm TCEP, pH 8.0). The target protein was eluted using elution buffer (50 mm Tris-HCl, 150 mm NaCl, 500 mm imidazole, 1 mm TCEP, pH 8.0). The eluted fractions were then concentrated and further purified by size-exclusion chromatography using a Superdex 200 column. The protein concentration was measured by Bradford protein assay with BSA as standard. Protein purity and molecular weight were determined by standard SDS-PAGE and LC-MS analysis. 0.05 µg of CrEspC was incubated with 5 µL (0.2 µg) mucus for 2 h, 8 h and 24 h at 37°C. Different amounts of CrEspC (0.0025, 0.0125, 0.05 µg) were incubated with 5 µL (0.2 µg) ALI-derived mucus for 2 h, followed by the visualization of degraded products as described above.

## Results

### ALI-cultured colonoid monolayers produce a physiologically relevant mucus layer

**Figure 1.**
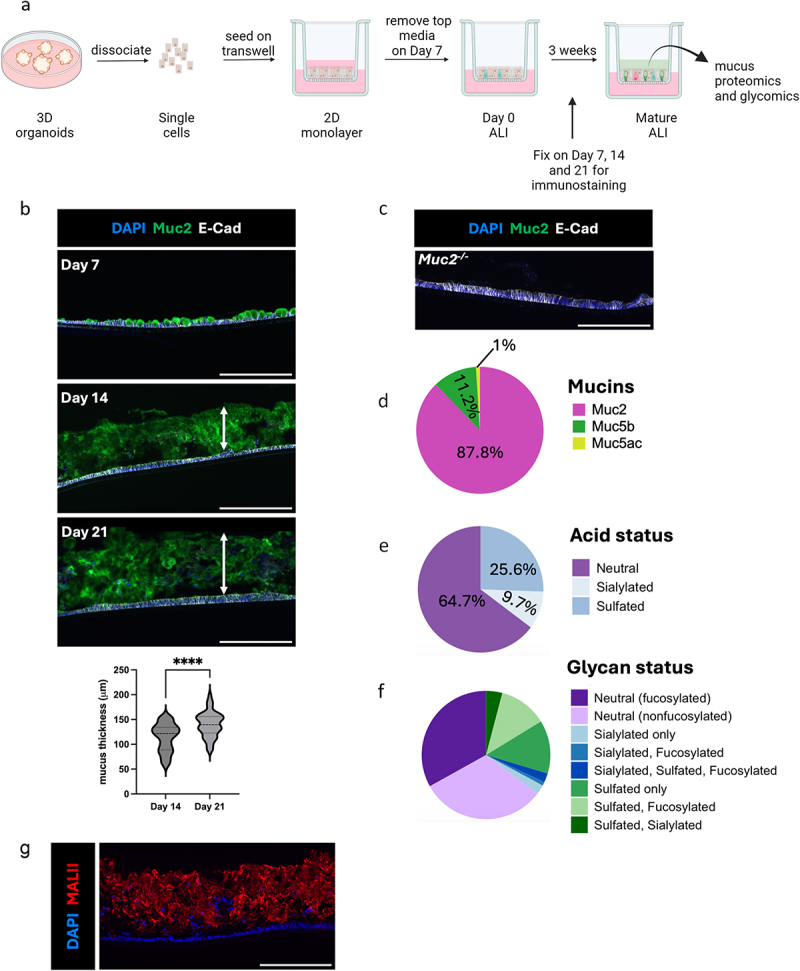
The ALI model produces a physiologically relevant mucus layer. a, The experimental procedure to generate ALI-derived mucus, created with Biorender.com. b, The formation of the mucus layer after growing ALI cultures for 7 days, 14 days and 21 days. Arrows indicate the mucus layer. The average mucus thickness at 14 days and 21 days is 114 ± 28 µm, and 140 ± 25 µm, respectively. c, Immunostaining of *Muc2*^*-/-*^ ALI (at 21 days). ALI cross-sections were stained with DAPI (blue), anti-Muc2 (green) and anti-E-cadherin (white). d, Proteomics analysis of gel-forming mucins present in mouse ALI-derived mucus. e-f, Pie chart of acidic (e) and various subsets (f) of mucin-type O-glycans in colonic ALI-derived mucus. g, MALII (red) staining of *Muc2*^*+/+*^ ALI culture. Scale bar, 200 µm.

Organoid-derived ALI monolayers have been previously used to generate mucus,^34^ but the dynamics of their mucin secretion and the composition of the resulting mucus have not been defined. To create ALI cultures, we grew 3D colonoids from the distal colons of Muc2^+/+^mice and generated 2D monolayers by subculturing the 3D colonoid-derived single cells on transwells for 1 week, followed by removal of the apical media (Day 0 of ALI). Cells were then maintained under ALI conditions for an additional 1 to 3 weeks (Figure 1(a)). The monolayers were fixed on days 7, 14 and 21 of ALI culturing, followed by immunostaining for Muc2. At day 7, the ALI monolayers displayed abundant Muc2 +ve goblet cells but showed no overlying mucus layer (Figure 1(b)). In sharp contrast, ALI monolayers showed extensive Muc2 staining and a gradually thickening mucus layer, from day 14 to day 21, reaching an average mucus thickness of 140 ± 25 µm. However, when colonoids from *Muc2*^*-/-*^ mice were used to grow ALI monolayers, no Muc2^+^ staining or mucus layer was detected (Figure 1(c)), confirming the key role of Muc2 in the ALI-derived mucus layer formation.

We next collected mucus on day 21 of ALI culture, and examined its protein composition using a timsTOF Pro ion mobility mass spectrometer. Of the total gel-forming mucin proteins detected, the majority (87.8%) were Muc2 (Figure 1(d)). The remaining mucin was found to be Muc5b (11.2%) and Muc5ac (1%), both of which are also present in *in vivo* colonic mucus.^46,47^ Moreover, a number of proteins previously recovered from mouse colonic mucus were identified in the ALI-derived mucus, including Fc fragment of IgG-binding protein (Fcgbp), Kallikrein-1 (Klk1), Calcium-activated chloride channel regulator 1 (Clca1), Trefoil factor family 3 (Tff3) and Zymogen granule membrane protein 16 (Zg16) (Supplemental Fig. S1A), all of which are produced by goblet cells.^46,48^ ALI-derived mucus was also found to contain various antimicrobial peptides, including members of the regenerating protein family (Reg3β and Reg4), galectin (Galectin-3, 4 and 6), and defensins (α-defensin 2, defensin-9, 23 and 30) (Supplemental Fig. S1B), mimicking the *in vivo* situation.^49,50^ Overall, the proteomics data demonstrate the complex nature of the secreted mucus in the ALI system.

We next assessed the glycosylation pattern of the secreted mucus. ALI-derived mucus collected on day 21 was reduced to release O-linked glycans, and structurally characterized by LC-MS as previously described.^42,51^ Three main classes of glycans, including sialylated (9.7%), sulfated (25.6%) and neutral (64.7%) glycans, and their subclasses were identified (Figure 1(e,f)), similar to those identified in mucus isolated directly from the mouse colon.^42^ Moreover, when the ALI-derived mucus was stained with *Maackia amurensis* lectin II (MALII, recognizing α2,3-linked sialylated and sulfated glycans), it was MALII positive (MALII^+^) (Figure 1(g)), matching a typical feature of the mucus produced by goblet cells in the murine distal colon.^52^

Taken together, these data demonstrate that ALI colonoid monolayers produce a mucus layer that is highly similar and physiologically relevant to *in vivo* colonic mucus.

### *ALI-derived mucus protects the underlying IECs from* C. rodentium *infection*

Since colonic mucus is known to prevent luminal microbes from reaching the underlying epithelium *in vivo*, we sought to test the ability of *C. rodentium* to infect the ALI monolayers.

**Figure 2.**
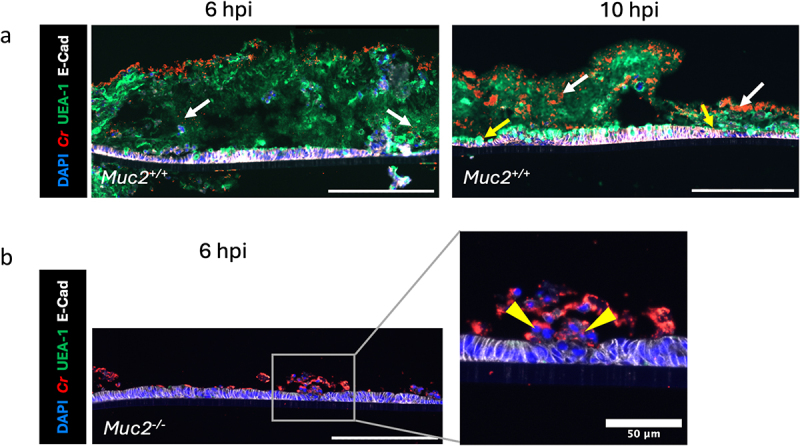
The ALI model is able to recapitulate pathogen-mucus interaction *in vivo*. a, *C. rodentium* infection of *Muc2*^*+/+*^ ALI cultures for 6 h and 10 h. Scale bar, 200 µm. b, *C. rodentium* infection of *Muc2*^*-/-*^ ALI culture for 6 h. ALI cross-sections were stained with anti-*C. rodentium* LPS (red), Ulex europaeus agglutinin-1 (UEA-1, green), DAPI (blue) and E-cadherin (white). Scale bar, 200 µm. White arrows indicate *C. rodentium* present in the mucus. Yellow arrows indicate *C. rodentium* close to IECs. Yellow arrowheads indicate cell sloughing.

We grew ALI monolayers from the distal colons of *Muc2*^*+/+*^ and *Muc2*^*-/-*^ mice for 21 days, and infected them with wild type (WT) *C. rodentium* at an MOI of 25. To ensure the infection was evenly distributed, *C. rodentium* was resuspended in a small volume (30 μL) of DMEM and carefully layered onto the surface of the mucus, with infected cultures fixed at different time points. To visualize *C. rodentium* interactions with the mucus and IECs, fixed cultures were immunofluorescently stained with anti-LPS (*C. rodentium*) and Ulex Europaeus Agglutinin I (UEA-I, a lectin that binds to fucosylated glycoproteins [i.e., mucins]). At 6 hours post-infection (6 hpi), the majority of *C. rodentium* remained on the surface of the mucus covering the *Muc2*^*+/+*^ ALI monolayers, with only a small number of *C. rodentium* detectable within the mucus layer (Figure 2(a), white arrows). By 10 hpi, a significant fraction of the *C. rodentium* (white arrows) had penetrated into the mucus layer, along with modest signs of mucus disruption. Occasionally, *C. rodentium* (yellow arrows) was found close to the underlying IECs. In contrast, when *Muc2*^*-/-*^ ALI monolayers (lacking a mucus layer) were infected, most *C. rodentium* were found in close proximity to IECs, even at 6 hpi, in concert with extensive IEC damage and sloughing (Figure 2(b), yellow arrowheads). To further illustrate the importance of mucus production in protecting the IECs from *C. rodentium* infection, we also infected 2D submerged IEC monolayers with *C. rodentium*. The 2D submerged monolayers derived from the colons of *Muc2*^*+/+*^ and *Muc2*^*-/-*^ mice were poorly differentiated and did not produce mucus (Supplemental Fig. S2). As a result, they were equally susceptible to *C. rodentium* infection. These findings demonstrate that ALI monolayers, but not submerged monolayers, produce mucus protecting the underlying IECs from *C. rodentium* infection.

### *Exposure to sialic acid accelerates* C.rodentium *infection of ALI colonoid monolayers*

**Figure 3.**
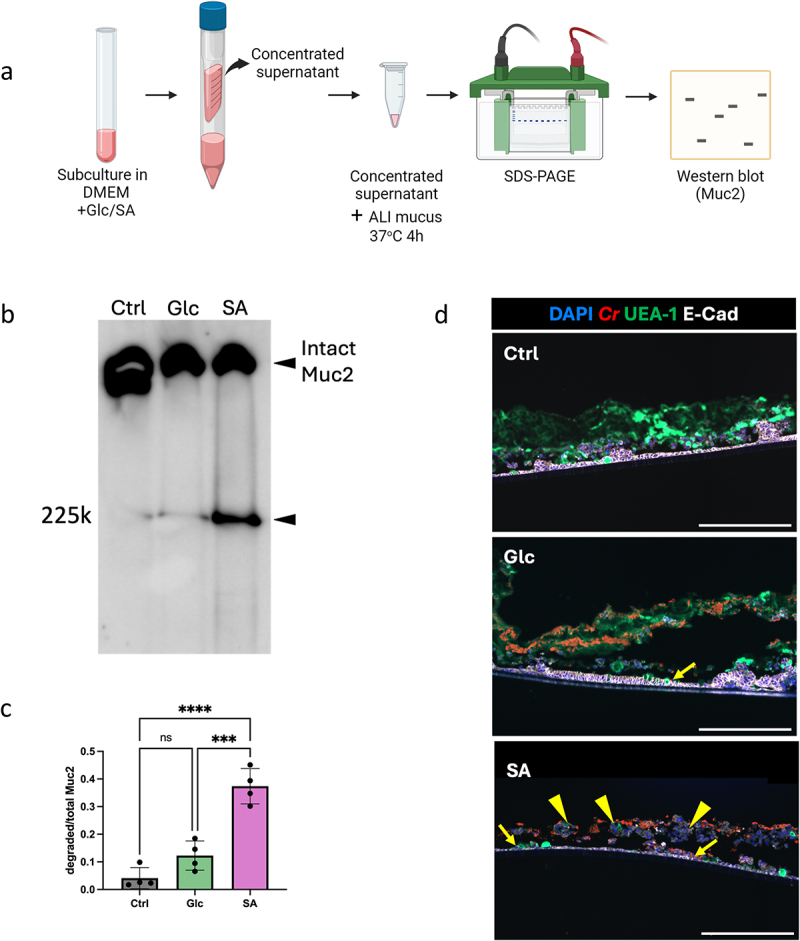
Sialic acid enhances *C. rodentium*’s ability to degrade ALI-derived mucus and infect IECs. a, Diagram of mucus degradation assay *in vitro*, created with Biorender.com. b, Mucus degradation assay using the supernatant from *C. rodentium* grown in media containing 0.45% glucose (Glc) or sialic acid (SA). Mucus incubated with *C. rodentium* supernatants was loaded onto 3–8% Tris-acetate gels and run through electrophoresis. Proteins were visualized by western blot using an anti-Muc2 antibody. c, Quantification analysis of degraded Muc2 band to total Muc2 band, ***, *p* < 0.001; ****, *p* < 0.0001. d, Immunostaining of ALI cultures infected with *C. rodentium* in the presence of glucose or sialic acid. ALI cross-sections were stained with anti-*C. rodentium* LPS (red), UEA-1(green), DAPI (blue) and E-cadherin (white). Yellow arrows indicate *C. rodentium* close to IECs. Yellow arrowheads indicate cell sloughing. Scale bar, 200 µm.

We previously determined that exposure to the monosaccharide sialic acid enhances the ability of *C. rodentium* to degrade BSM, as well as to adhere to the CMT-93 murine IEC line.^26^ We tested whether sialic acid would also enable *C. rodentium* (and its secreted proteins) to degrade ALI-derived mucus more effectively. *C. rodentium* was pre-cultured in media containing 0.45% sialic acid (or glucose as a control), and its proteins secreted in the supernatants were concentrated and then incubated with the ALI-derived mucus. Degraded mucin bands were separated by SDS-PAGE gels and probed using an anti-Muc2 antibody (Figure 3(a)). The supernatant from sialic acid-preconditioned *C. rodentium* supernatant induced greater mucus degradation, as indicated by the more intense Muc2 band at a molecular weight of 225kDa (Figure 3(b), and Supplemental Fig. S3A), as compared to the supernatant from glucose-preconditioned *C. rodentium*. Indeed, the ratio of the intensity of the degraded Muc2 band to that of the total Muc2 band was significantly higher under sialic acid-preconditioned conditions than that under glucose-preconditioned conditions (Figure 3(c)).

We next examined whether sialic acid would facilitate *C. rodentium* infection of ALI colonoid monolayers. *C. rodentium* was pre-cultured in the same media as described above, followed by its direct addition onto the ALI monolayers at an MOI of 25 for 10 hours. Glucose-preconditioned *C. rodentium* was able to penetrate the ALI-derived mucus, and a small number of bacteria (yellow arrow) reached the IECs (Figure 3(d)). In contrast, *C. rodentium* preconditioned in sialic acid was found to almost completely degrade the mucus layer, as evidenced by very limited UEA-1 staining. In addition, numerous *C. rodentium* were found to be adherent to the IECs (yellow arrow), along with widespread IEC cell sloughing, indicated by yellow arrowheads (Figure 3(d)). Interestingly, while infection with sialic acid-preconditioned *C. rodentium* led to significant mucus degradation, it also induced increased gene transcription of *Muc2* within the infected monolayers (Supplemental Fig. S3B). These findings suggest that goblet cells may undergo heightened *Muc2* transcription as a compensatory response to active mucus degradation by *C. rodentium* secreted proteins. This response could relate to the increased Muc2 secretion detected after *C. rodentium* infection in mice.^11^

Together, our results demonstrate that sialic acid promotes *C. rodentium*-mediated degradation of ALI-derived mucus and infection of the underlying IECs.

### C. rodentium’s *ability to degrade ALI-derived mucus depends on EspC*

**Figure 4.**
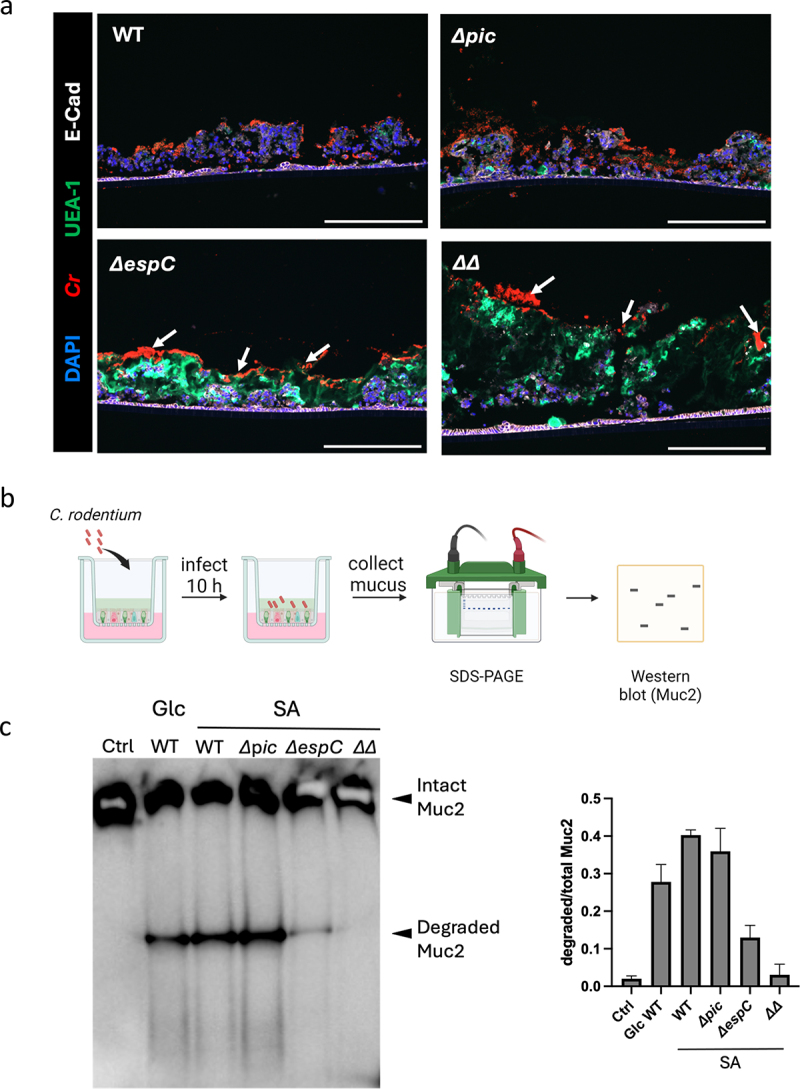
A comparison of the ability of WT, *Δpic, ΔespC* and *ΔpicΔespC C. rodentium* strains to infect mouse ALI cultures and to degrade the ALI-derived mucus. a, The ALI cultures were infected with different *C. rodentium* strains, including WT, *Δpic, ΔespC* and *ΔpicΔespC* double mutant (*ΔΔ*). ALI cross-sections were stained with anti-*C. rodentium* LPS (red), UEA-1(green), DAPI (blue) and E-cadherin (white). White arrows indicate *C. rodentium* on the top of the mucus. Scale bar, 200 µm. b, Diagram of mucus degradation assay after *C. rodentium* infection, created with Biorender.com. ALI cultures were infected with different bacterial strains, followed by the collection of mucus from infected cultures. c, mucus from infected ALI was collected and run on a 3%-8% Tris-acetate gel, followed by western blot using an anti-Muc2 antibody.

We previously showed that exposure to sialic acid enhances *C. rodentium*’s ability to degrade BSM, and that this reflected the actions of Pic, a class II SPATE known to exhibit mucinolytic activity.^43,53^ *C. rodentium* also secreted another serine protease autotransporter EspC. As a class I SPATE, EspC has not been previously shown to degrade mucins. To define the mechanisms by which sialic acid promotes *C. rodentium*-driven degradation of ALI-derived mucus and its ability to infect underlying IECs, we infected ALI monolayers with WT *C. rodentium* and its derivative strains, including *pic* mutant (*Δpic*), *espC* mutant (*ΔespC*), and *ΔpicΔespC* double mutant (*ΔΔ*) strains. At 10 hpi, ALI monolayers were fixed in 4% PFA and immunostained for *C. rodentium* LPS and mucus (UEA-1). Despite Pic’s previously demonstrated role in degrading BSM, the *Δpic* strain behaved in a similar fashion to WT *C. rodentium*, causing almost complete loss of the ALI-derived mucus layer (Figure 4(a)). This was accompanied by a heavy infection of the underlying IECs, and widespread cell sloughing. In contrast, the *ΔespC* and *ΔΔ* strains were found to remain on the surface of the mucus layer (white arrows), rarely penetrating the mucus, and causing little if any damage to the ALI monolayers. To confirm that these phenotypes reflected differences in Muc2 degradation, we collected samples from the ALI cultures that were infected with different *C. rodentium* strains, and examined Muc2 degradation patterns by Western blot (Figure 4(b)). As expected, uninfected samples showed a single large intact Muc2 band. Notably, the intensity of this band was significantly reduced in WT and *Δpic* infected samples, in concert with the presence of a strong degraded Muc2 band (Figure 4(c)). In contrast, the intact Muc2 band detected in the *ΔespC* and *ΔΔ* infected samples were much stronger, similar to that in uninfected samples. While the *ΔespC* sample showed a very weak degraded Muc2 band, no Muc2 degradation was observed in the *ΔΔ* infected mucus (Figure 4(c)). These findings suggest that *C. rodentium* infection induces an EspC-dependent degradation of ALI-derived mucus.

To confirm the role of *C. rodentium* EspC in ALI-derived mucus degradation, we collected supernatants from WT, *Δpic*, *ΔespC*, and *ΔΔ* strains grown in DMEM (supplemented with 0.45% sialic acid), and incubated them with the ALI-derived mucus. The mucus incubated with WT and *Δpic* strain supernatants showed significant degradation (Supplemental Fig. S4A). In contrast, the degradation of ALI-derived mucus by the *ΔespC* supernatant was minimal, and was completely missing when the *ΔΔ* supernatant was tested. Interestingly, when we repeated this experiment using BSM which shows high similarity to human MUC19,^54^ we saw significant degradation by WT as well as the *ΔespC*, whereas the *Δpic* and *ΔΔ* strains failed to degrade BSM (Supplemental Fig. S4B). Taken together, we confirm that *C. rodentium* uses Pic to degrade BSM, whereas it largely relies on EspC to degrade ALI-derived mouse colonic mucus.

### *EspC expressed in* E.coli *DH5α degrades ALI-derived mucus*

**Figure 5.**
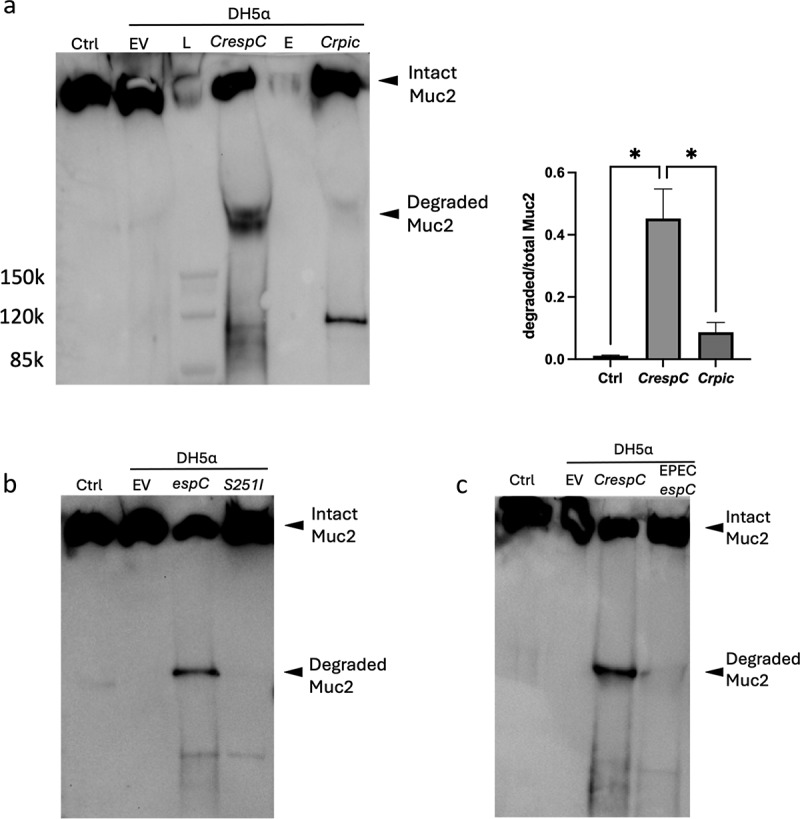
Mucus degradation assay using protein secreted by SPATEs expressing *E. coli* DH5α. a, The degradation of mouse ALI-derived mucus by supernatant from *E. coli* DH5α expressing *CrespC* or *Crpic*. EV, empty vector; L, protein ladder; E, empty lane. Note, the anti-Muc2 antibody shows cross-activity against Pic (~110 kDa). *, *p* < 0.05. b, The degradation of mouse ALI-derived mucus by supernatant from *E. coli* DH5α expressing *CrespC* or *CrespC-S251I*. c, The degradation of mouse ALI-derived mucus by supernatant from *E. coli* DH5α expressing *CrespC* or EPEC*espC*. Samples from the mucus degradation assay were reduced and run on a 3%-8% Tris-acetate gel, followed by western blot using an anti-C-terminus Muc2 antibody.

To further explore the different abilities of EspC and Pic to degrade ALI-derived mucus, we cloned the *C. rodentium espC* (*CrespC*) and *pic* (*Crpic*) genes downstream of an arabinose promoter into a pBAD30 vector, generating the plasmids pBAD30-*CrespC* and pBAD30-*Crpic*, respectively. These plasmids were transformed into *E. coli* strain DH5α, which possesses no EspC or Pic homologues, and has no known mucinase activity.^55,56^ *E. coli* DH5α strains carrying the pBAD30 empty vector (EV), pBAD30-*CrespC* or pBAD30-*Crpic* were cultured in the presence of 0.2% arabinose for 4 h to induce the expression of the respective autotransporters. The resulting supernatants were incubated with ALI-derived mucus overnight. As shown in Figure 5(a), no ALI-derived mucus (Muc2) degradation was detected by supernatant collected from *E.coli* DH5α containing EV. However, *E.coli* pBAD30-*CrespC* supernatant was able to induce significant Muc2 degradation. When *E.coli* pBAD30-*Crpic* supernatant was incubated with the mucus, the intensity of the degraded Muc2 band was significantly less, at approximately 19% of that seen by the *E. coli* pBAD30-*CrespC* supernatant.

Examining protein sequences, both EspC and Pic were found to share the conserved protease domain “GDSGS”, which was previously proven critical for the mucinolytic activity of Pic.^57^ To understand if this domain is also required for EspC to degrade the ALI-derived mucus, we performed site-directed mutagenesis of pBAD30-*CrespC*, changing the conserved serine in the protease domain GD*S*GS into an isoleucine (S251I).^25^ As a result of this mutation, *E.coli* pBAD30-*CrespC-S251I* supernatant failed to degrade mucus (Figure 5(b)), indicating that the GDSGS domain is required for the mucinolytic activity of EspC.

*C. rodentium* EspC shares 55.4% identity and 71.6% similarity with the EspC expressed by the clinically important pathogen EPEC (Supplemental Fig. S5). To address whether EPEC EspC also possesses mucinolytic activity, we cloned the EPEC *espC* gene into pBAD30, generating the plasmid pBAD30-EPEC*espC*. When *E. coli* pBAD30-EPEC*espC* supernatant was incubated with ALI-derived mucus, a degraded Muc2 band was observed, although its intensity was not as strong as that seen in *E. coli* pBAD30-*CrespC* supernatant treated mucus (Figure 5(c)). To verify the identity of the degraded Muc2 band, we performed Coomassie staining and excised the “degraded Muc2 band” for MS analysis. As Coomassie staining showed weak signals for the degraded Muc2 bands, we also confirmed these bands by western blot (Supplemental Fig. S6). Indeed, the top hit identified by MS analysis contains 9 Muc2-derived peptides (Table 1).

**Table 1.** List of Muc2-derived peptides identified in the degraded Muc2 band by MS.

Peptide number	Muc2 peptide sequences
1	VPVESYVR
2	TVVLLTDDKK
3	RSETPFAR
4	HETQEVQIK
5	QLVALPYK
6	FAPGYDVCVK
7	EGGSGIVCQPK
8	TEIVPGK
9	VPCSAVSVMK

Altogether, our results demonstrate that *C. rodentium* EspC, when heterologously expressed by *E. coli* DH5α possesses strong mucinolytic activity toward ALI-derived mucus.

### Recombinant CrEspC alone is sufficient to degrade ALI-derived mucus

**Figure 6.**
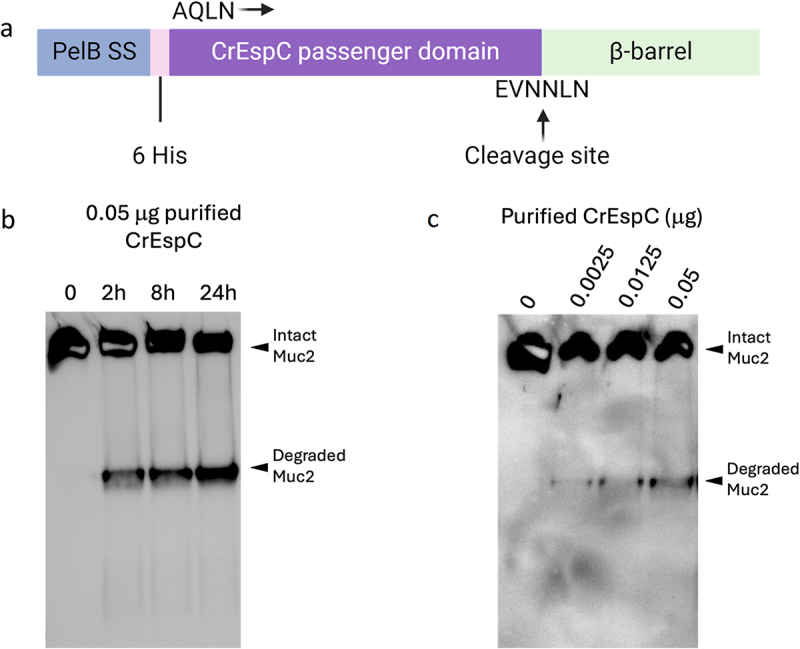
Recombinant CrEspC is able to degrade mouse ALI-derived mucus. a, The sequence information used to express recombinant CrEspC by *E. coli* BL21 Star (DE3) system, created with Biorender.com. SS, signal sequence. b, The degradation of mouse ALI-derived mucus by 0.05 µg purified CrEspC for 2 h, 8 h and 24 h. c, The degradation of mouse ALI-derived mucus by different amounts (0.0025, 0.0125, 0.05 µg) of purified CrEspC for 2 h. Samples were reduced and run on a 3%-8% Tris-acetate gel, followed by western blot using an anti-C-terminus Muc2 antibody.

The above findings demonstrate the mucinolytic activity of EspC, but it remains unclear whether *C. rodentium* EspC is able to degrade mucus (Muc2) independent of any other bacterial products. To address this question, we expressed CrEspC using the *E. coli* BL21 Star (DE3) and the PelB signal peptide.^58,59^ An N-terminal His tag was also added in front of the CrEspC passenger domain to allow column purification of the recombinant CrEspC protein (Figure 6(a)). 0.05 µg of recombinant CrEspC was incubated with mouse ALI-derived mucus for 2 h, 8 h and 24 h, followed by detection of the degraded Muc2 band by western blot. As shown in Figure 6(b), recombinant CrEspC was sufficient to induce Muc2 degradation within 2 h, and this degradation was more prominent after longer incubation times (8 h and 24 h). Additionally, when the same amount of mucus was incubated with increasing amounts of recombinant CrEspC (0.0025, 0.0125, 0.05 µg ) for 2 h, we observed a dose-dependent increase in Muc2 degradation, as revealed by the gradually increasing intensity of the degraded Muc2 band (Figure 6(c)). These findings demonstrate that recombinant CrEspC is sufficient to degrade mouse ALI-derived mucus on its own.

## Discussion

The exact mechanisms by which bacterial pathogens successfully infect the intestinal mucosal surfaces of their hosts are poorly defined as there are multiple defenses and barriers they must overcome, some of which, like mucus, are difficult to model. Here, we used an ALI colonoid monolayer model to better understand how enteric bacterial pathogens circumvent the colonic mucus layer. Our analysis revealed significant similarities between ALI-derived mucus and mucus collected directly from the mouse colon, including its protein composition, glycosylation pattern, and its protective role against *C. rodentium* infection.^11,42,48^ Moreover, we identified the class I SPATE EspC as an underappreciated mucinase that mediates the ability of *C. rodentium* to degrade ALI-derived mucus and thereby promote its pathogenesis.

While few studies have examined *C. rodentium* infection in culture, we first tested this pathogen on 2D submerged IEC monolayers generated from 3D mouse colonoids. However, as these submerged monolayers did not produce mucus, monolayers derived from the colons of *Muc2*^*+/+*^ and *Muc2*^*-/-*^ mice proved equally susceptible to *C. rodentium* infection. In contrast, the ALI colonoid monolayers derived from the colons of *Muc2*^*+/+*^ and *Muc2*^*-/-*^ mice were well differentiated due to the model’s oxygen enrichment,^31^ with the *Muc2*^*+/+*^ ALI monolayer producing a thick mucus layer, a feature completely lacking in the *Muc2*^*-/-*^ derived monolayers. It remains unclear whether there is a compensatory increase in other mucins, such as Muc5b and/or Muc5ac in *Muc2*^−/−^ organoids. Despite this, we were able to confirm the key role of Muc2 in providing protection against *C. rodentium* infection using the ALI system, consistent with its role *in vivo*.^11^ Indeed, we also found the glycosylation pattern and composition of ALI-derived mucus were highly similar to those found in the mucus *in vivo*. Moving forward, we anticipate that ALI organoid cultures will provide a useful tool to define how aspects of Muc2/IEC glycosylation and goblet cell function contribute to host defense against A/E pathogen infections.

Sialic acid is a terminal glycan found in the glycosylated mucin throughout the gut.^60^ Many studies have shown that commensal microbes use sialidases to remove sialic acid residues from glycosylated mucins, and this monosaccharide can be used to cross-feed other commensal microbes or be utilized by pathogens for expansion and infection.^15,61^ Notably, *C. rodentium* was reported to use sialic acid as a carbon source for growth and adaption to the host’s intestinal lumen. *C. rodentium* grown in the presence of sialic acid also secreted more SPATEs, EspC and Pic.^43^ Following upon our recent discovery, we now demonstrate that sialic acid increases *C. rodentium*’s degradation of ALI-derived mucus, accelerating its ability to attach to and disrupt underlying IECs.

We found that *C. rodentium*’s ability to degrade ALI-derived mucus was primarily due to EspC. Prior studies have attributed several roles to EspC, but as a class I SPATE, these have largely focused on cytotoxicity to host cells. As only class II SPATEs have so far been reported to exhibit mucus degrading activity,^19,24,62^ we decided to confirm the mucinase activity of EspC with two approaches. First, we expressed CrEspC in *E. coli* DH5α, a bacterial strain known to have no mucinase activity (also confirmed in our study, Figure 5(a)). We demonstrated that the supernatant of *E. coli* DH5α carrying the WT copy of *CrespC* (pBAD30-*CrespC*), but not the point mutant of *CrespC* (pBAD30-*CrespC-S251I*), was able to degrade the ALI-derived mucus. It should be noted that S251I is within the serine protease motif GD*S*GS in both EspC and Pic. Interestingly, the supernatant of *E. coli* DH5α carrying EPEC *espC* (pBAD30-*EPECespC*) also exhibited mucinase activity toward the mouse ALI-derived mucus, albeit with less activity as compared to CrEspC. While it is tempting to speculate that this may contribute to the limited ability of EPEC to infect mouse colons,^3,63^ further studies are required. As a second approach to confirm the mucinase activity of EspC, we used an *E. coli* BL21(DE3) expression system to purify recombinant CrEspC. We found that recombinant CrEspC was sufficient to degrade the ALI-derived mucus in a dose-dependent manner. Taken together, these data demonstrate that CrEspC functions as a mucinase and is able to degrade mouse ALI colonoid-derived mucus.

Consistent with the key role of EspC in degrading mucus, *C. rodentium ΔespC* was significantly less capable of degrading ALI-mucus layers and infecting the underlying IECs than the WT strain. Notably, EspC was first identified in EPEC and shown to exert enterotoxic effects on intestinal tissues.^55,64^ EPEC EspC has also been demonstrated to regulate pore formation mediated by the T3SS.^65^ After translocation into epithelial cells, EPEC EspC leads to cell detachment and cell death by cleaving fodrin, paxillin, focal adhesion kinase (FAK) and pro-caspase 3.^66,67^ Complementing these studies, our findings reveal that at earlier stages of infection, *C. rodentium* secretes EspC as a means to cleave the host’s protective mucus layer, facilitating pathogen access and attachment to the underlying IECs.

The finding that CrPic exhibits significantly lower mucinase activity toward ALI-derived mucus as compared to CrEspC was unexpected. Previous studies have shown that Pic, expressed by several different bacterial pathogens, can efficiently degrade BSM,^57^ hog gastric mucin (HGM),^56,57^ mucus produced by the human LS174T IEC line^68^ and submerged monolayer-secreted mucus.^30^ Moreover, our previous study suggests that *C. rodentium* culture supernatant causes BSM degradation through the actions of Pic.^43,53^ In contrast, EspC is unable to degrade BSM.^19,43^ These studies suggest that the ability of EspC and Pic to degrade mucins likely depends on the sources of mucins examined. Despite the mucin substrate differences, CrEspC and CrPic appear to have a synergistic effect on the degradation of ALI-derived mucus.

In summary, our results highlight the robustness of ALI colonoid monolayers as a model to uncover the molecular basis of pathogen-mucus interactions. We demonstrated that *C. rodentium* was able to penetrate the ALI-derived mucus primarily via EspC-mediated mucus (Muc2) degradation, rather than Pic, enabling its enhanced adherence to, and disruption of IECs. Notably, we have revealed for the first time that EspC, originally classified as a class I SPATE, exhibits a typical feature of class II SPATEs, i.e., mucinolytic activity, allowing it to cleave ALI-derived mucus.

## Supplementary Material

Supplemental Material

## Data Availability

The mass spectrometry proteomics data have been deposited to the ProteomeXchange Consortium via the PRIDE^69^ partner repository with the dataset identifiers PXD060170 for the proteomics data of ALI-derived mucus (https://proteomecentral.proteomexchange.org/cgi/GetDataset?ID=PXD060170.) and PXD061145 for the proteomics data of degraded ALI-derived mucus by CrEspC (https://proteomecentral.proteomexchange.org/cgi/GetDataset?ID=PXD061145).
