# Supplementary material for: Defining enteric bacterial pathogenesis using organoids: Citrobacter rodentium uses EspC, an atypical mucinolytic protease, to penetrate mouse colonic mucus
Source: Gut Microbes. 2025 May 5;17(1):2494717. doi: 10.1080/19490976.2025.2494717 (PMC12054374; doi:10.1080/19490976.2025.2494717)

Supplemental Figure

Supplemental Figure 1. Proteomics analysis on A) Mucus-associated proteins, and B) antimicrobial peptides present in mouse ALI-derived mucus.


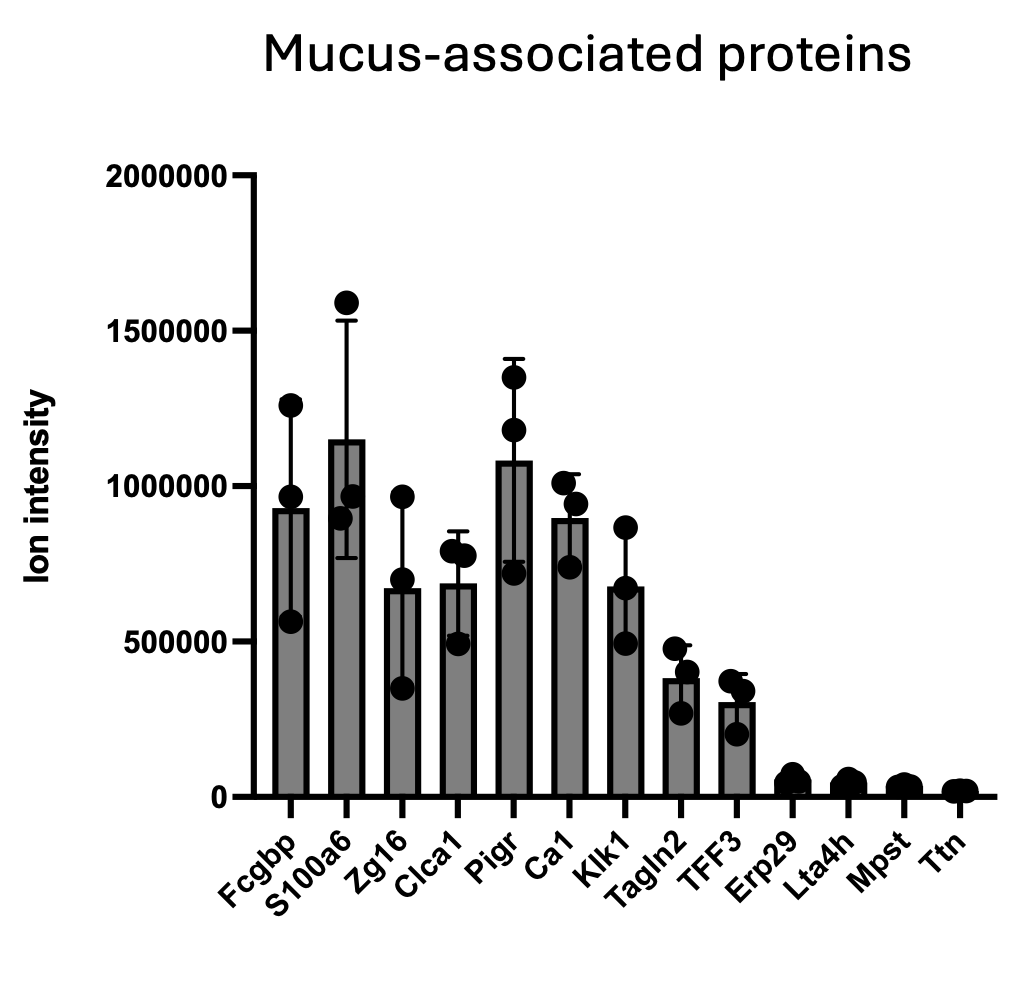


A


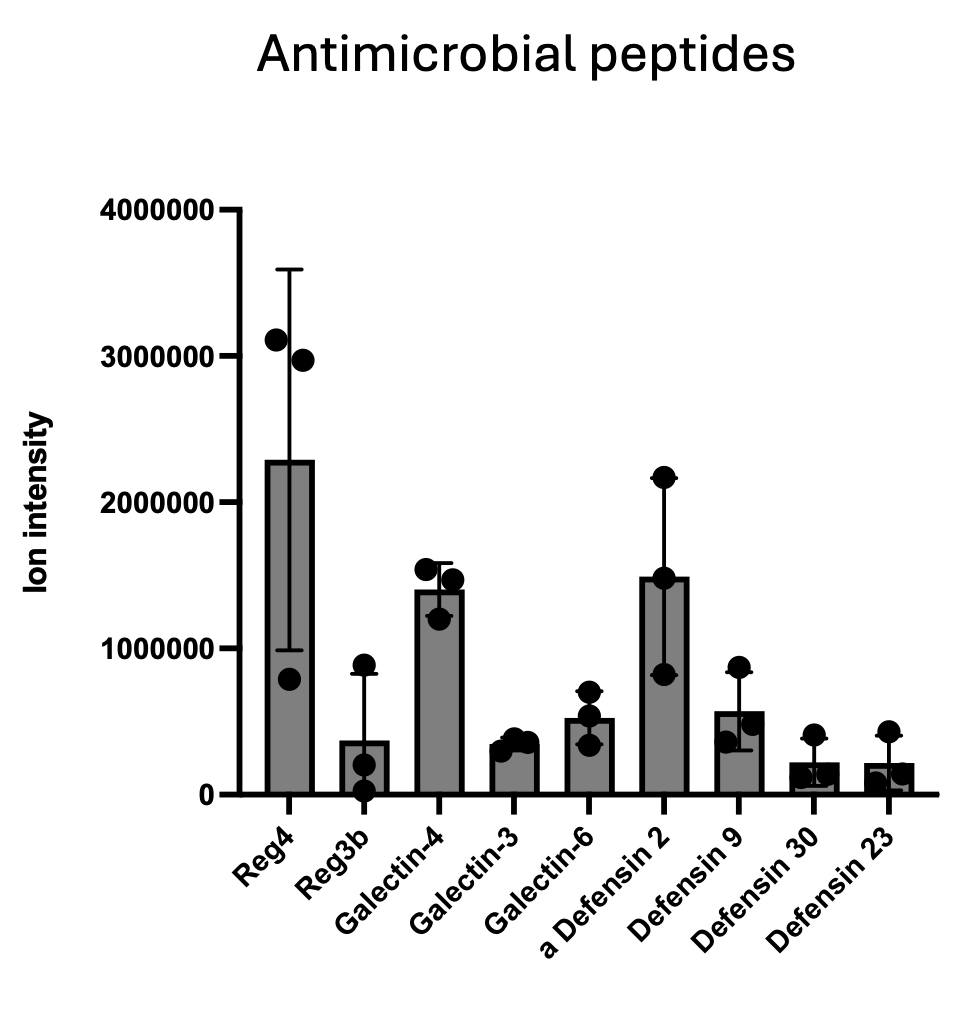


B

Supplemental Figure 2. *C. rodentium* infection of primary cell-derived monolayers. A, 6-hour infected submerged monolayers were stained with *C. rodentium* LPS (red), DAPI (blue), UEA-1 (green) and phalloidin (white). B, comparison of heavily infected cells between *Muc2*^+/+^ and *Muc2*^-/-^ monolayer.

B

A


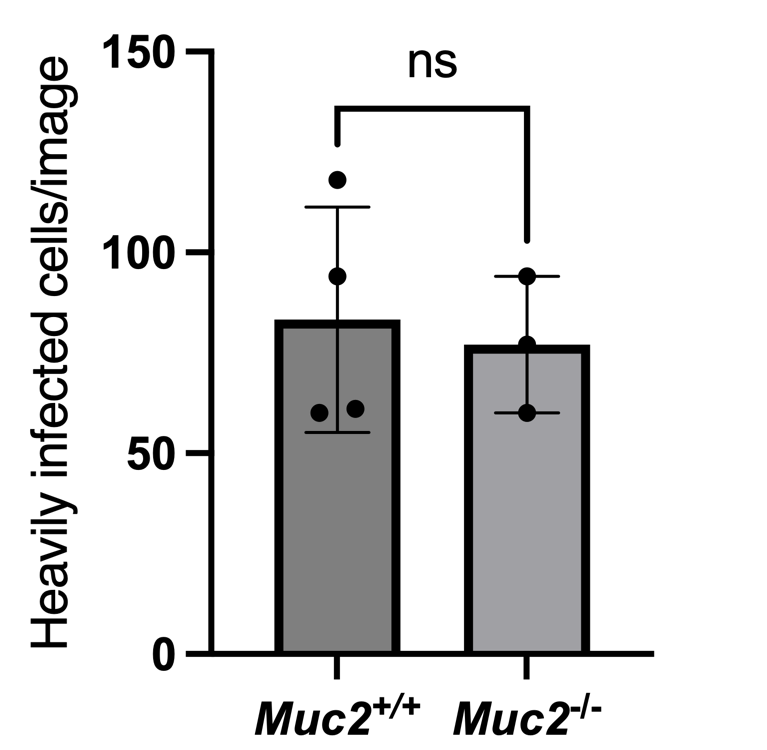

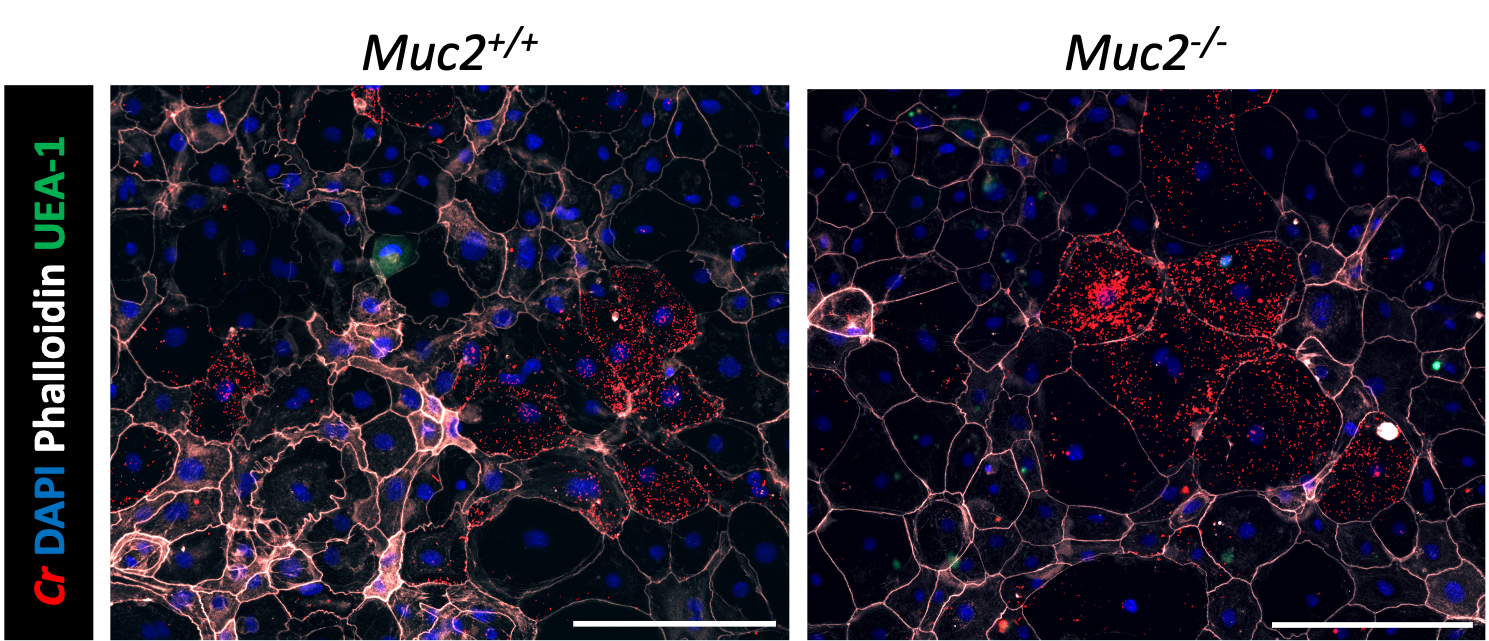


B

Supplemental Figure 3. *C. rodentium* infection of primary cell-derived monolayers. A, 6-hour infected submerged monolayers were stained with *C. rodentium* LPS (red), DAPI (blue), UEA-1 (green) and phalloidin (white). B, comparison of heavily infected cells between *Muc2*^+/+^ and *Muc2*^-/-^ monolayer.


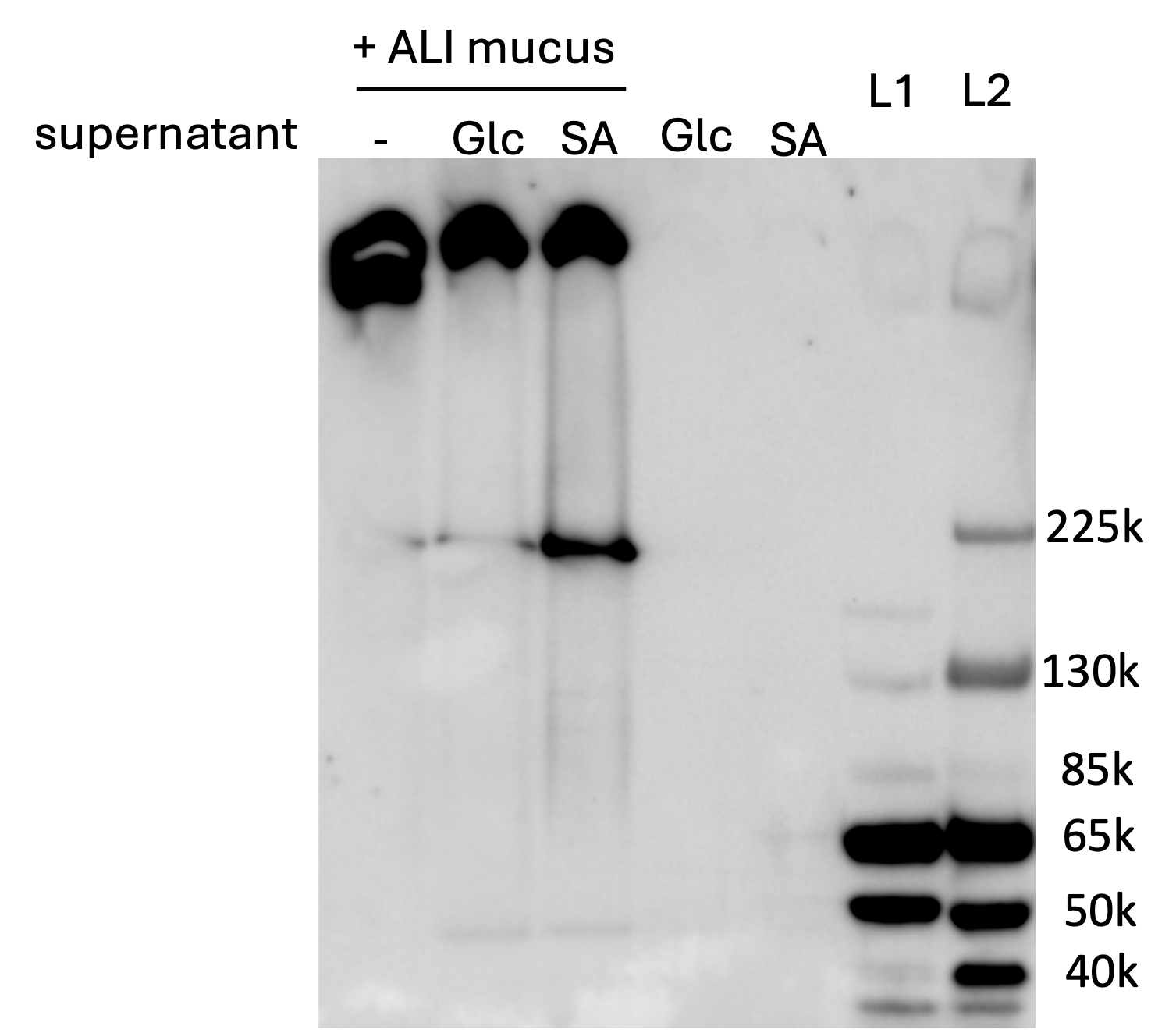

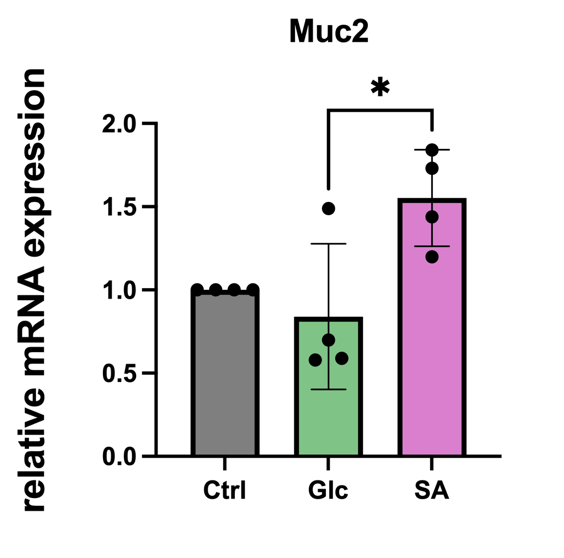


B

A

Supplemental Figure 4. *C. rodentium* infection of primary cell-derived monolayers. A, 6-hour infected submerged monolayers were stained with *C. rodentium* LPS (red), DAPI (blue), UEA-1 (green) and phalloidin (white). B, comparison of heavily infected cells between *Muc2*^+/+^ and *Muc2*^-/-^ monolayer.

A

B


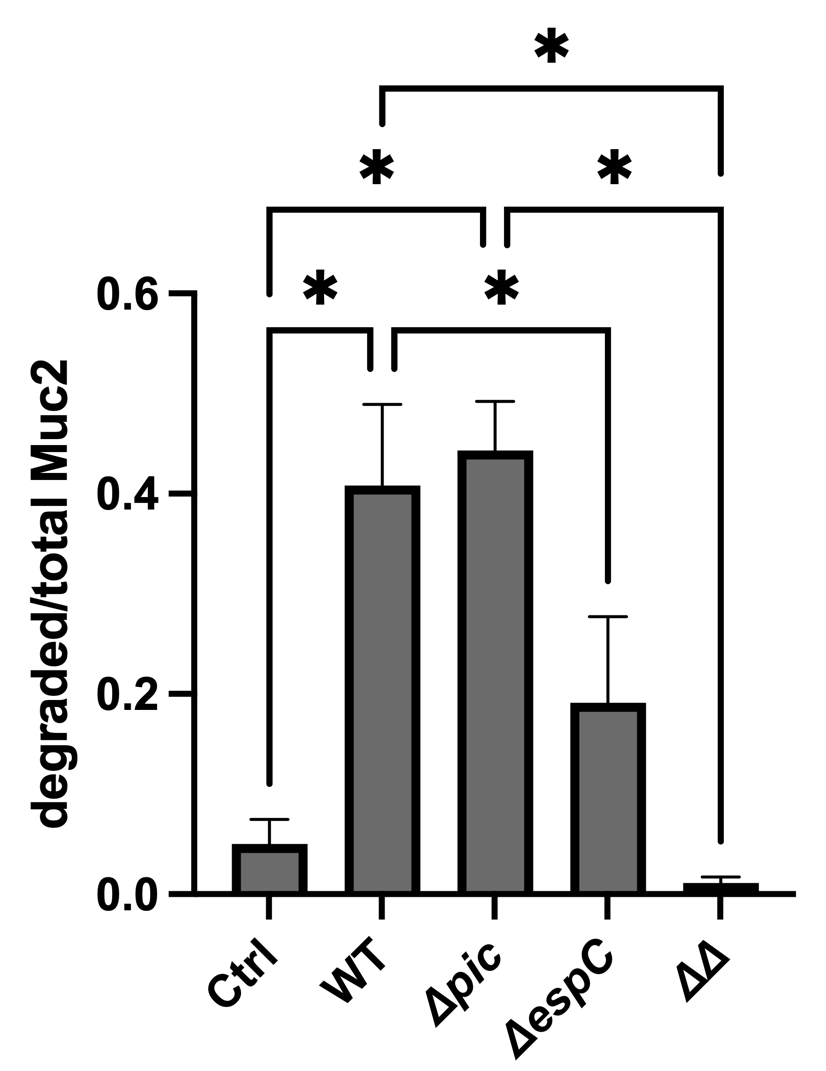

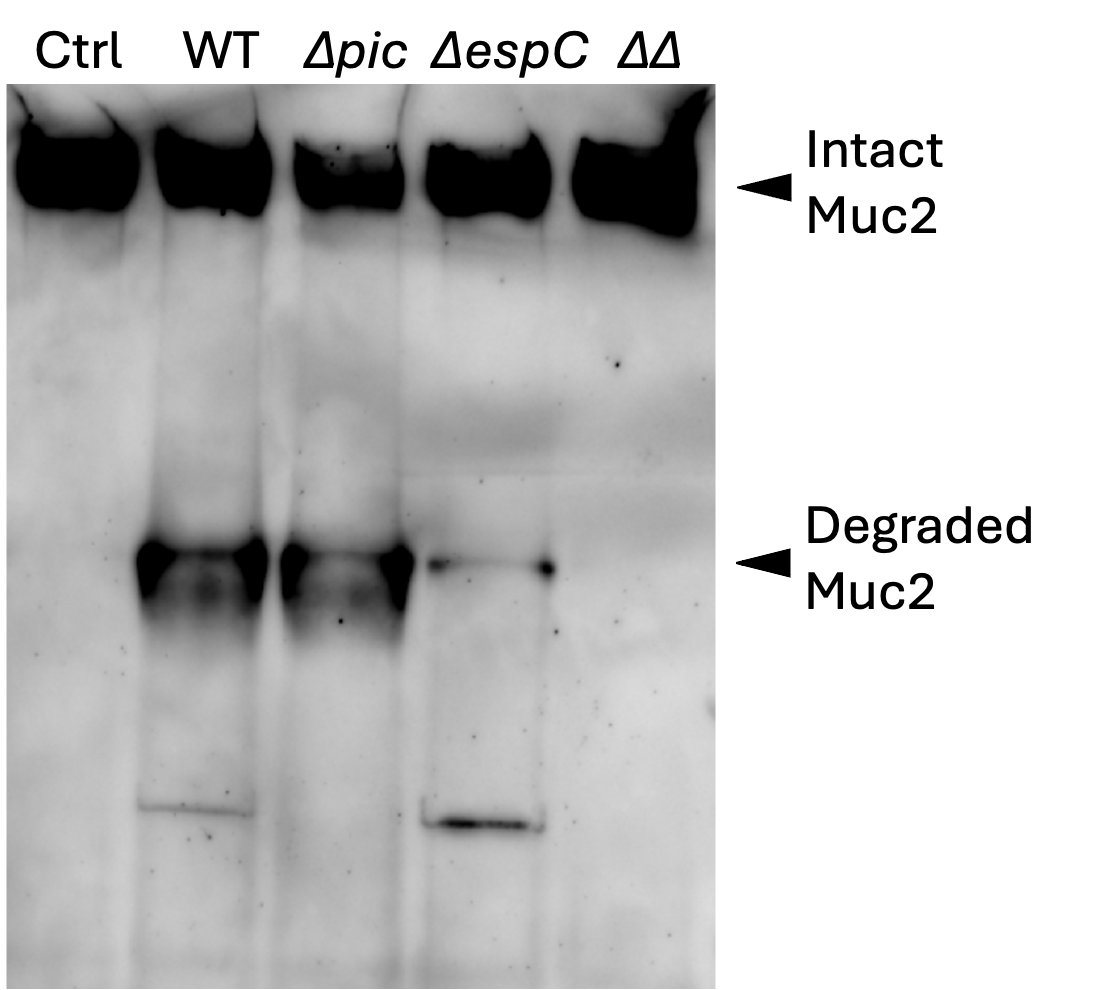


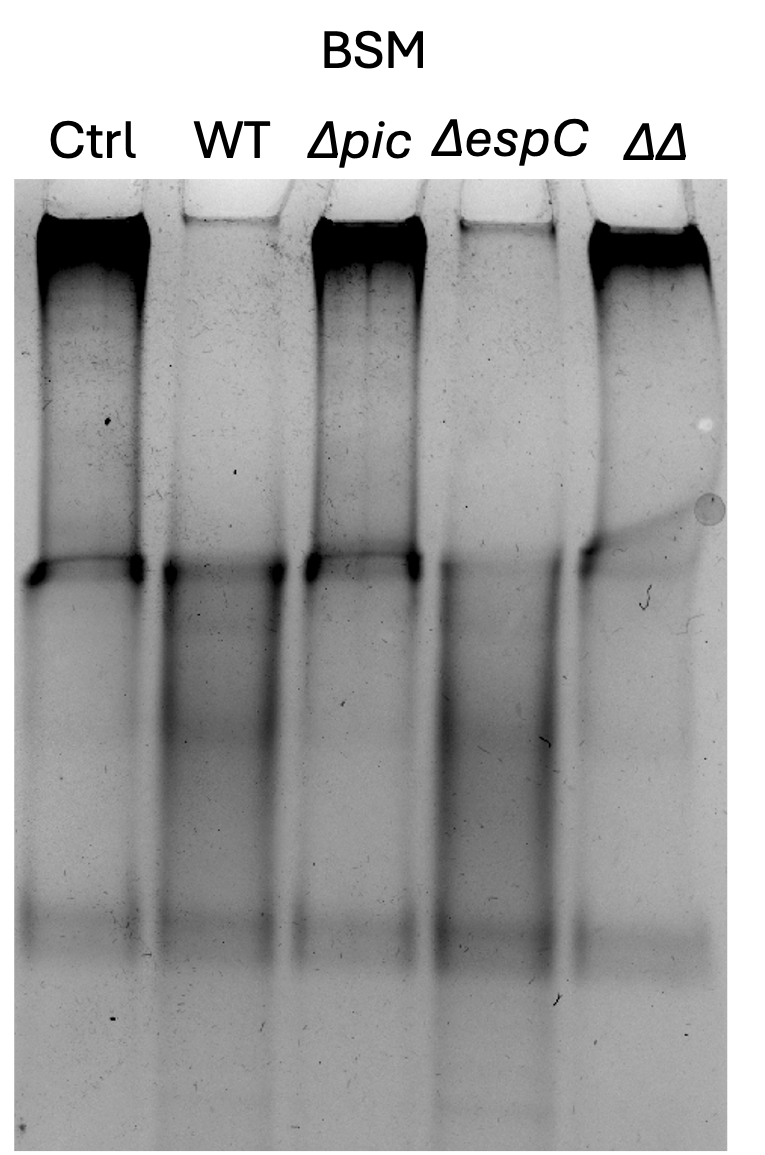


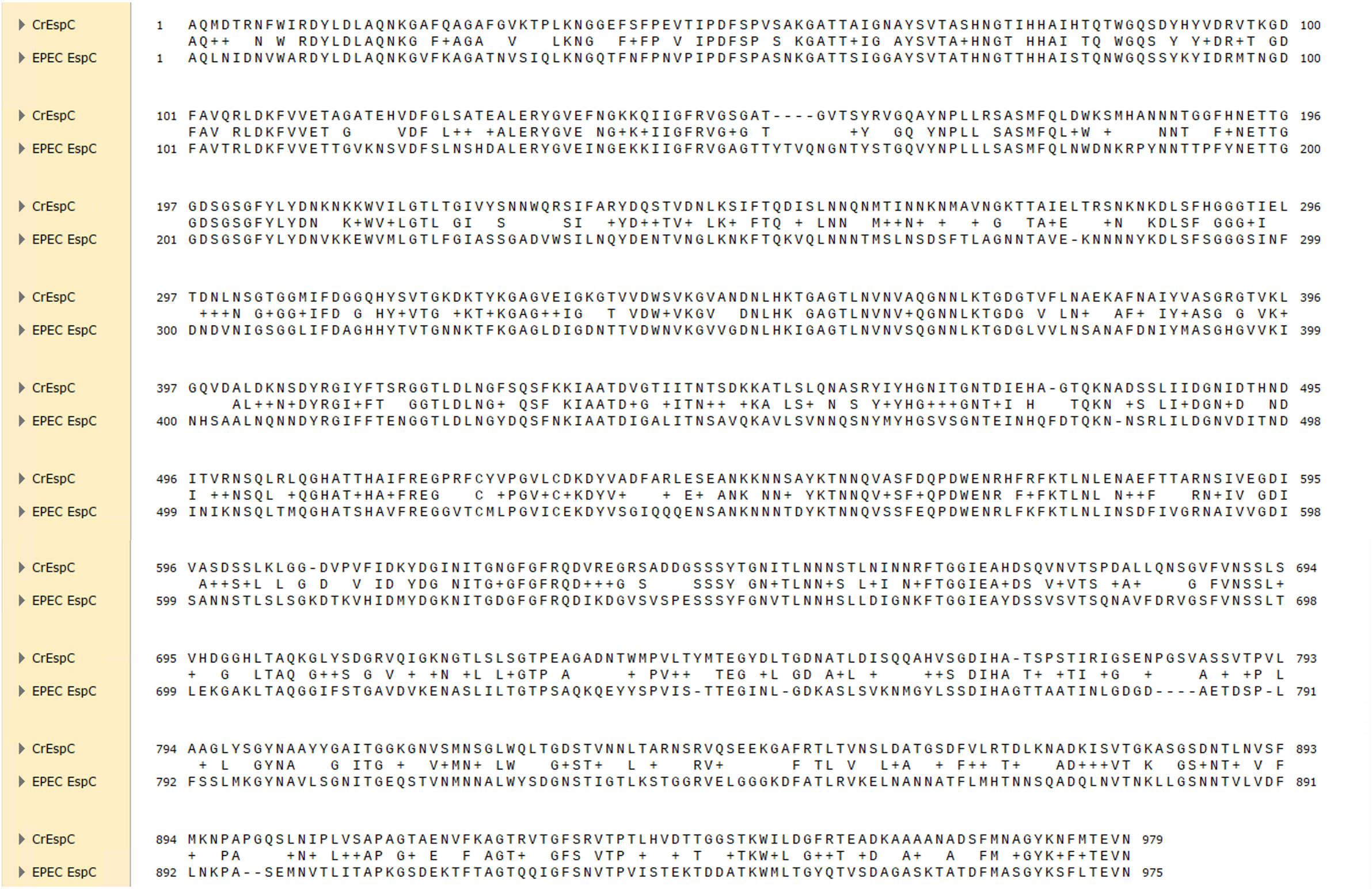
Supplemental Figure 5. Alignment of passenger domains of CrEspC and EPEC EspC.

Supplemental Figure 6. Degraded Muc2 band prepared for MS analysis. Western blot using anti-C-terminus Muc2 confirmed the mucus degradation. The area corresponding to the degraded Muc2 (rectangle) on the gel was excised and proteins were isolated and analyzed by MS. EV, empty vector.


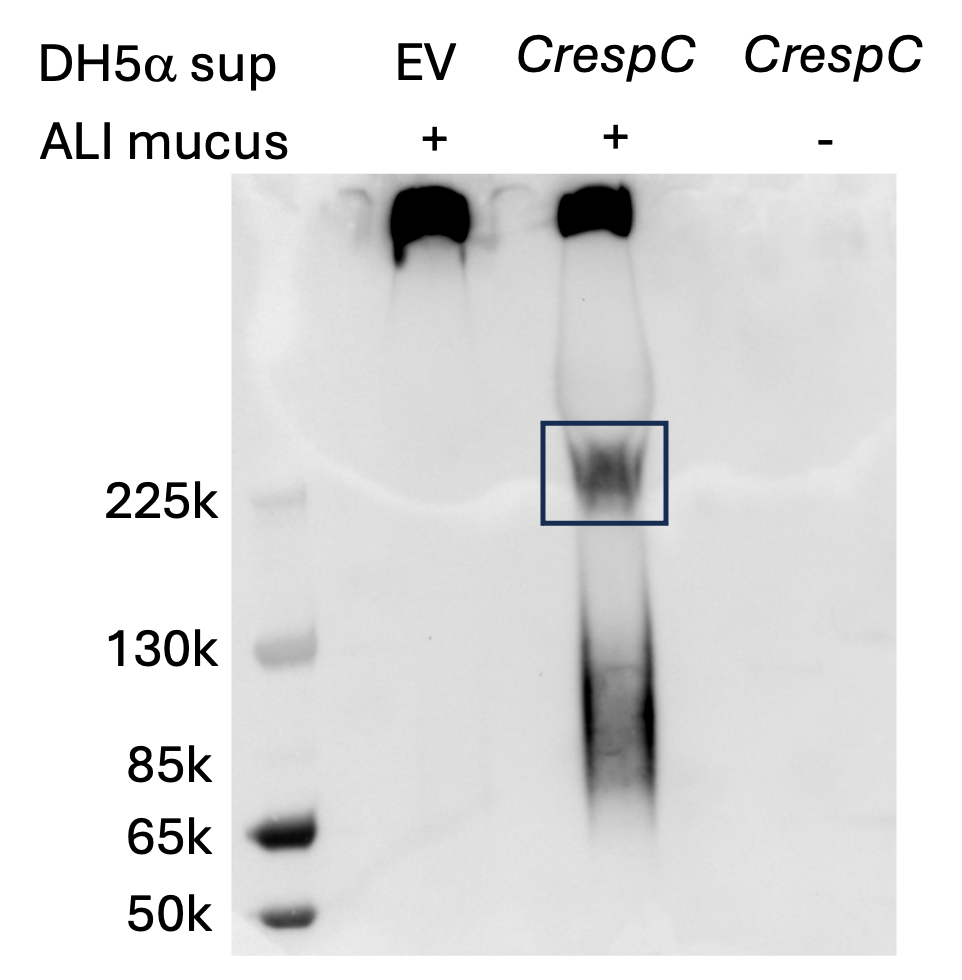

Supplement: Supplemental Material [file KGMI_A_2494717_SM8089.docx]
